# A consensus statement on perinatal mental health during the COVID-19 pandemic and recommendations for post-pandemic recovery and re-build

**DOI:** 10.3389/fgwh.2024.1347388

**Published:** 2024-02-21

**Authors:** Leanne Jackson, Mari Greenfield, Elana Payne, Karen Burgess, Munira Oza, Claire Storey, Siân M. Davies, Kaat De Backer, Flora E. Kent-Nye, Sabrina Pilav, Semra Worrall, Laura Bridle, Nina Khazaezadeh, Daghni Rajasingam, Lauren E. Carson, Leonardo De Pascalis, Victoria Fallon, Julie M. Hartley, Elsa Montgomery, Mary Newburn, Claire A. Wilson, Joanne A. Harrold, Louise M. Howard, Jane Sandall, Laura A. Magee, Kayleigh S. Sheen, Sergio A. Silverio

**Affiliations:** ^1^ Department of Psychology, Institute of Population Health, Faculty of Health and Life Sciences, University of Liverpool, Liverpool, United Kingdom; ^2^ Faculty of Wellbeing, Education and Language Studies, The Open University, Milton Keynes, United Kingdom; ^3^ Department of Women & Children’s Health, School of Life Course & Population Sciences, Faculty of Life Sciences & Medicine, King’s College London, London, United Kingdom; ^4^ Petals: The Baby Loss Counselling Charity, Cambridge, United Kingdom; ^5^ The Ectopic Pregnancy Trust, London, United Kingdom; ^6^ International Stillbirth Alliance, Bristol, United Kingdom; ^7^ School of Psychology, Faculty of Health, Liverpool John Moores University, Liverpool, United Kingdom; ^8^ Centre for Research in Psychology and Sport Sciences, Health and Wellbeing Research, The University of Hertfordshire, Hatfield, United Kingdom; ^9^ HELIX Service, Maternal Mental Health Services, King’s College Hospital NHS Foundation Trust, London, United Kingdom; ^10^ Chief Midwifery Office, NHS England—London Region, London, United Kingdom; ^11^ Maternity Services, Guy’s and St. Thomas’ NHS Foundation Trust, London, United Kingdom; ^12^ Section of Women’s Mental Health, School of Mental Health & Psychological Sciences, Institute of Psychiatry, Psychology & Neuroscience, King’s College London, London, United Kingdom; ^13^ Research Development, UK Biobank, Manchester, United Kingdom; ^14^ Division of Methodologies, Department of Midwifery, Florence Nightingale Faculty of Nursing, Midwifery & Palliative Care, King’s College London, London, United Kingdom; ^15^ South London and Maudsley NHS Foundation Trust, London, United Kingdom; ^16^ School of Social Sciences, College of Health, Science and Society, University of the West of England Bristol, Bristol, United Kingdom

**Keywords:** consensus statement, COVID-19, perinatal mental health, women’s health, recommendations for policy and practice

## Abstract

**Introduction:**

The COVID-19 pandemic posed a significant lifecourse rupture, not least to those who had specific physical vulnerabilities to the virus, but also to those who were suffering with mental ill health. Women and birthing people who were pregnant, experienced a perinatal bereavement, or were in the first post-partum year (i.e., perinatal) were exposed to a number of risk factors for mental ill health, including alterations to the way in which their perinatal care was delivered.

**Methods:**

A consensus statement was derived from a cross-disciplinary collaboration of experts, whereby evidence from collaborative work on perinatal mental health during the COVID-19 pandemic was synthesised, and priorities were established as recommendations for research, healthcare practice, and policy.

**Results:**

The synthesis of research focused on the effect of the COVID-19 pandemic on perinatal health outcomes and care practices led to three immediate recommendations: what to retain, what to reinstate, and what to remove from perinatal mental healthcare provision. Longer-term recommendations for action were also made, categorised as follows: Equity and Relational Healthcare; Parity of Esteem in Mental and Physical Healthcare with an Emphasis on Specialist Perinatal Services; and Horizon Scanning for Perinatal Mental Health Research, Policy, & Practice.

**Discussion:**

The evidence base on the effect of the pandemic on perinatal mental health is growing. This consensus statement synthesises said evidence and makes recommendations for a post-pandemic recovery and re-build of perinatal mental health services and care provision.

## 1 Introduction

The COVID-19 pandemic presented an unprecedented health system shock to the world between January 2020 and May 2023. Although first detected in Wuhan, China, on 31 December 2019 (1), the virus—a respiratory disease with high mortality risk for individuals with pre-existing comorbidities (2)—spread quickly, worldwide. Concerns about the mortality and spread of the novel virus prompted a global, co-ordinated implementation of social and physical distancing restrictions. Meanwhile, research efforts turned towards vaccine development (3) and understanding the health system shock and the possible ramifications for short-, medium-, and long-term health, especially as the world braced itself for the further pandemic of mental health issues caused by the virus and associated fears, bereavements, and restrictions (4). Maternity care was significantly disrupted during government-mandated lockdown restrictions (5). Social and physical distancing restrictions interrupted access to routine maternity care and adversely impacted perinatal mental health (6, 7) and child development (8, 9). Worryingly, these restrictions saw increased instances of child neglect, child abuse, and domestic abuse risk (10); restricted access to reproductive healthcare including abortion services (11); increase in maternal morbidity (12); and serious adverse obstetric events such as stillbirths (13, 14). Further, the potential for maternity staff to experience work-related trauma and subsequent post-traumatic stress disorder (PTSD) was likely to have been exacerbated beyond levels already recognised as significant (15–17). The extent of the long-term impacts of the pandemic, however, has yet to be fully realised and may take years to be understood completely.

This article presents a consensus statement on amassed evidence from research and syntheses on perinatal mental health undertaken during the COVID-19 pandemic. We suggest recommendations in the form of what healthcare policy, services, and professionals should retain, reinstate, and remove from their care provision in the immediate period of post-pandemic recovery and re-build. We also provide guidance on longer-term recommendations for practice.

## 2 Methods

**Figure 1:**
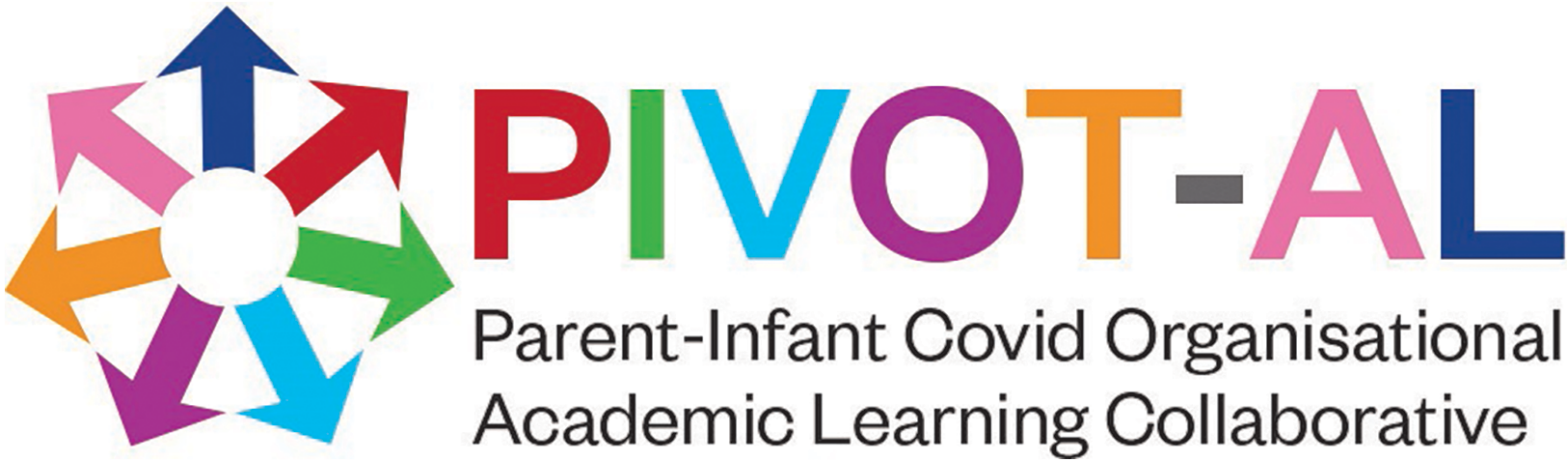
The PIVOT-AL logo.

This consensus statement was originally conceived by a collective of cross-disciplinary researchers (Psychologists, Psychiatrists, Sociologists, Anthropologists, Midwives, Obstetricians, Obstetric Physicians, Physiologists, and Patient Advocates; mainly based in London and Liverpool, UK) who, in late 2020/early 2021 wanted to synthesise evidence from research they had conducted during the early stages of the pandemic about how it had affected perinatal mental health outcomes, services, and care. They secured funding from the Society for Reproductive and Infant Psychology—via a Research Development Workshop Grant (ref:- SRIP/DWA/01)—to do so, which contributed to the second origin—a policy-oriented research dissemination event held at The Royal Society of Medicine (The RSM) in London on 22 September 2022. The RSM event was hosted by PIVOT-AL, a national collaborative in the UK of over 60 researchers, academics, policymakers, and members of third sector organisations from more than 25 institutions (see Figure 1). During the pandemic, the collaborative undertook research focused on the impact of the pandemic on maternal, child, and family health, healthcare professionals, and service provision. A formal synthesis of this evidence on perinatal mental health was presented as a key part of the programme at The RSM event. This consensus statement provides a summary of this evidence and identifies priorities for future research, policy, and healthcare practice.

A recognised approach for deriving consensus statements is usually to construct a panel of experts amongst whom ideas are shared with a focus on establishing priorities for research, healthcare practice, and policy (18). Discussions at this event were based on the expert knowledge of attendees and enhanced by patient and public involvement and engagement (PPIE) at both the event and in writing the statement. The cross-disciplinary nature of the group allowed for a breadth and depth of perspectives to be represented. The authors recognise that whilst this synthesis is extensive, it is not exhaustive of all the research efforts which took place in perinatal mental health services across the UK during the COVID-19 pandemic. Neither does it have a reach into global literature—which is equally important, but would be inappropriate to incorporate as part of an assessment into UK policy and practice. Therefore, this statement does not aim to provide a comprehensive nor systematic review of the literature base, but rather represents an overview of issues and priorities discussed by attendees at the dissemination event. Indeed, the statement presents the consensus reached by academics and clinical experts who authored the literature included in the synthesis and by those present at the dissemination event.

## 3 Available evidence

The perinatal mental health research captured by The PIVOT-AL National Collaborative primarily focused on post-partum mental health and the transition into new motherhood during the COVID-19 pandemic. However, extensive efforts have also spanned the psycho-social experiences of pregnancy and childbirth, incidences of domestic abuse and violence, and support requirements of perinatal mental health staff and services during mandated social and physical distancing restrictions.

One of the earliest PIVOT-AL investigative efforts was The Pregnancy and Motherhood Study [PRaM; (19)]. A large, online, national survey was distributed to pregnant and post-partum women during initial mandated lockdown restrictions (20), during the initial easing of social distancing restrictions (21), and post-“Freedom Day” [defined as the easing of all legal restrictions on social contact; (22)]. The PRaM Study involved the distribution of a battery of psychometric measures (19, 23), with nested qualitative interviews in accordance with the corresponding mandated lockdown restrictions (24, 25).

Quantitative findings indicated that 43% and 61% of post-partum women were experiencing clinically relevant levels of depression and anxiety symptoms, respectively (19). Perceived psychological change, resulting from the introduction of social distancing measures, predicted unique variance in the risk of clinically relevant maternal depression (30%) and anxiety symptoms (33%), respectively (19). These data were consistent with UK data found in global comparisons of perinatal mental health data as reported by a consortium of the RISEUP-PPD Network, where the UK consistently ranked highly amongst reports of increased symptoms of perinatal anxiety and depression (26). The PRaM Study also rapidly developed and validated a research short form of the Postpartum Specific Anxiety Scale for use in global crises [PSAS-RSF-C (23);]. This short form was translated into Chinese, Dutch, French, Italian, and Spanish (23), and validations are underway including in Persian [PSAS-IR-RSF-C; (27)].

Qualitatively, the PRaM Study found post-partum women continued to experience distress throughout the pandemic, despite the easing of social distancing restrictions (24). A lack of support for the schooling of older children was particularly inflammatory to maternal mental health and wellbeing disturbance (24). Antenatally, respondents were consistent across timepoints in feeling their pregnancy was overshadowed by uncertainties pertaining to the pandemic, which left respondents grieving for the loss of the kind of transition to motherhood that they would have had in the absence of mandated lockdown restrictions (25).

Echoing these findings, an analysis of qualitative data from women recruited to The King's Together Fund Changing Maternity Care Study identified tensions between good and poor practices, which affected perinatal psycho-social wellbeing (28). Results included dyadic pairs of experiences as women struggled to navigate the uncertainties of the pandemic and pregnancy, alone. The dyadic pairs included the following: “lack of relational care vs. good practice persisting during the pandemic”; “denying the embodied experience of pregnancy and birth vs. trying to keep everyone safe”; and “removed from support network vs. importance of being at home as a family” (28). Consistent with other PIVOT-AL works, the realities of maternity care were disappointing compared with expectations and experiences before the pandemic, which exacerbated distress (28). The lack of access to relational care, the introduction of telemedicine and reliance on virtual appointments, and the exclusion of partners from routine care were particularly challenging for emotional wellbeing. This was despite an acknowledgement of the pressures placed on healthcare professionals and on NHS services during the unprecedented times of the pandemic (28). A lack of access to emergency and gynaecological care was also flagged as being detrimental to the care of early pregnancy loss and later perinatal deaths (29, 30).

A critical review and mapping of service provision suggested that perinatal distress had increased, which was attributable to the increasing inaccessibility of support services (31). However, this was occasionally countered by services providing reconfigured and/or extended perinatal mental health services. As healthcare transitioned from pandemic to para-pandemic circumstances, it was imperative to provide support for perinatal mental health professionals within the context of developing new post-pandemic services (31). Some women struggled to engage with virtual mental health assessments in perinatal mental health services (32). This was especially concerning for circumstances whereby virtual appointments prevented disclosure of urgent needs and risks, e.g., in cases of domestic abuse (32). However, for women who struggled with the practicalities of attending face-to-face consultations, e.g., due to travel time, virtual appointments offered a flexible and well-received alternative (32).

Maintaining perinatal mental health services was found to be challenging for ethnic minority women, who experienced many difficulties and disruptions in accessing perinatal mental healthcare, which exacerbated pre-existing challenges such as living in insecure social housing and experiencing financial hardship (33). Most had a strong preference for face-to-face consultations and experienced high levels of social isolation and heightened anxiety as the pandemic continued (33). A large study was also conducted that utilised linked maternity and mental health records held within the Early Life Cross-Linkage in Research (eLIXIR) database (34). Data from three NHS Foundation Trusts (including one Mental Health Trust) in South London constitute the eLIXIR database (34, 35). Research using an interrupted time series study design found that the rate of recording domestic abuse and violence during national lockdown restrictions was reduced by 78% in mental healthcare settings. There was also an increased prevalence of positive screening on the Whooley depression screening measure, by 40%, in the same period (35).

A large body of international work investigating the effects of the pandemic on new, expectant, and bereaved parents [COCOON; (36)] is underway, complete with a nested qualitative study [PUDDLES; (30)] which focuses on the experiences of women bereaved by pregnancy loss (e.g., early elective abortion, pregnancy of unknown location, miscarriage, ectopic pregnancy, molar pregnancy, or termination of pregnancy due to foetal anomaly) or perinatal death (stillbirth and neonatal death). Results specifically linked to the mental health outcomes are pending, but they will provide important insight into another aspect of perinatal mental health, not otherwise covered by the information synthesised above.

Whilst there has been much evidence to support worsening conditions for perinatal mental healthcare and support during the pandemic, the ending of the global health crises allows a period of reflection and reset for recovery and re-build from the health system shock. What follows are recommendations for immediate action, followed by long-term recommendations for policy, service provision, and research.

## 4 Discussion of recommendations

### 4.1 Immediate action

#### 4.1.1 What to retain

Access to essential reproductive services such as contraception and abortion (37, 38), ensuring high levels of relational care are prioritised in healthcare service and delivery (28, 31), and redoubling efforts to ensure that perinatal and infant mental health are given the parity of esteem of physical health concerns (39) are recommended for retention in line with other calls for prioritisation of specialist women's mental healthcare (40–42). Communication of health messaging to families should continue to be clear, concise, and consistent, and the option for remote care provision should be maintained (32, 33). However, this should be offered in line with clinical decision-making around safety and appropriateness for individual women and birthing people.

#### 4.1.2 What to reinstate

At a system level, reinstating time for processing and reflection on new directives for service delivery, as well as including the voices of healthcare professionals and service users, is important across all aspects of healthcare serving perinatal women (43). This will enable teams to consider how best to implement new service provisions. Bi-directional communication amongst central NHS management, individual trusts, and healthcare professionals is recommended to optimise satisfaction with care and workplace satisfaction for staff (31). Within this, the voices of perinatal women and birthing people must also be heard and their perspectives on prospective changes must be sought. Recommendations are also made to reinstate the autonomy and judgement of healthcare professionals in providing empathic, evidence-based care, including professional judgement on when to use remote vs. in-person care (32, 43).

During the pandemic, a large proportion of healthcare professionals were displaced within their services to provide support to COVID-19 wards (28), and early pregnancy and/or gynaecological services were dramatically rationalised (29). Maternity care was consequently stripped of vital service provision by specialist midwives for mental health and bereavement care (31). Evidence from the PIVOT-AL collaborative highlights the importance of protecting healthcare professionals across all aspects of perinatal care services from redeployment to ensure that a full complement of staff is available to perinatal women/people, their babies, and their families (31). This also requires recognising the importance of quality, holistic, post-partum care, specifically in the community (33). To re-establish these priorities, face-to-face care and support should be reinstated (24, 25, 35) and should remain the dominant form of care provision.

Finally, re-introducing consented partners, family members, and/or other trusted support (e.g., friends and Doulas) should be prioritised across all interactions across the perinatal period (28, 30, 31). Importantly, this form of support should be seen as part of the caregiving team and not simply visitors, and should be regarded as a basic birthing right, never again to be removed.

#### 4.1.3 What to remove

First, recommendations are made to cease blanket or “one size fits all” policies from being rolled out across all services without consideration of variations in demographic need or accessibility to essential support services (33), as this would lead to inequitable health services. During the pandemic, ethical, moral, and relational care was replaced by priorities of infection control (28, 31), thereby swapping a broad notion of safety that encompassed women's psychological safety for one bearing a narrow definition focused on the notion that safety was synonymous with not spreading the infection, and prioritising prevention of COVID deaths above other serious and potentially fatal risks such as severe mental health episodes, domestic abuse and violence, and suicide.

At this time, personalised care was often deprioritised (24, 25). Considering these findings, recommendations are made to cease the provision of exclusively virtual or remote care (28) and the exclusion of wanted birth partners (31). Furthermore, confusing and conflicting messaging amongst government organisations, Royal Colleges, individual NHS Trusts, and other Learned Academies has been a persistent issue of concern (28). When national public health messaging is necessary, disinformation and/or conflicting information must be stopped as a matter of utmost importance (24, 25). Messaging must be consistent from policy to practice, and policymakers and healthcare professionals must be agile enough to interpret and implement change in a uniform way.

### 4.2 Long-term recommendations

#### 4.2.1 Equity and relational healthcare

Equitable, relational care should be offered to all in the perinatal community (39), with special consideration made for populations who struggle to access healthcare (e.g., women from ethnic and sexual minority groups or those living with high levels of social complexity or in areas with high levels of social deprivation), who may be particularly avoidant of using perinatal mental health services (33). Support for women, birthing people, and their families should be curated, based on personalised needs assessments in circumstances of high physical, mental, or social risk (24, 25). It would also be prudent to not only maintain focus on the health of women and birthing people, but also attend to the established relationship amongst parental, child health, and wider family health, acknowledging the reciprocal nature of the caregiver-infant mental health outcomes (44) and ensuring healthcare professionals are working holistically (31) and with the whole family to be proactive and to intervene before families reach crisis point (45). We must also give greater energy to and focus on those families who find care hard to access (46); experience high levels of social complexity, inequality, and deprivation (47); may have a rooted distrust for the NHS and wider social care systems (48); or are generally underserved by the health and care system (49). In doing so, we must integrate psychological support across the healthcare systems linked to maternal and child health, especially for families who experience pregnancy losses (50), those whose babies are born premature or become ill (51), or whose babies die (30, 52), as these parents and families require additional psychological support as they access other parts of the healthcare system such as Neonatal Intensive Care Units [NICU; (30)] or perinatal bereavement care services (53).

#### 4.2.2 Parity of esteem in mental and physical healthcare with an emphasis on specialist perinatal services

Protecting healthcare professionals’ emotional wellbeing and capacity, protecting against redeployment, and arguing for a greater representation of minoritised staff are recommended across perinatal mental health services (31, 32), echoing broader calls across all maternity and children's healthcare services (54). A better integration of physical and mental healthcare is also required (39), whilst retaining and improving specialist perinatal mental health services (41). Community, educative, and public health engagement needs targeting to better support marginalised and disadvantaged communities suffering from perinatal mental health problems (33, 35). New and evolving information about the potential negative effects on perinatal mental health, transmitted from leading experts, should be concise, credible, and transparent (24, 25).

#### 4.2.3 Horizon scanning for perinatal mental health research, policy, and practice

Perinatal mental health research covers a broad expanse of time (preconception to post-partum), engages women and their families, and involves many aspects of the healthcare system. The ability to mobilise research using innovative methods and having prompt access to accurate, identifiable routine data is imperative for rapid-response research. The effects of the pandemic on mental health during preconception (55), after an early elective abortion or termination of pregnancy due to foetal anomaly, or following an early pregnancy loss or late perinatal death (30, 36), have yet to be fully understood and should remain areas of priority. Global data may also be useful to understanding best practices in aspects of perinatal mental healthcare that could be applied to the UK NHS context.

## 5 Conclusion

Post-partum distress was elevated during mandated social distancing restrictions imposed during the COVID-19 pandemic (19, 26). Qualitative and critical review literature contextualised these findings. Specifically, perinatal women struggled to navigate scaled-back maternity care and felt that their experience of maternity had been overshadowed by uncertainties and health anxiety pertaining to the pandemic (24, 25, 28, 31, 33). For families facing additional adversities (e.g., those experiencing domestic abuse and violence), the depletion of face-to-face care proved a particularly grievous threat to wellbeing (35). Finally, The Postpartum Specific Anxiety Scale—Research Short Form was produced and validated in English for use in global crises (23), allowing for a rapid assessment of post-partum anxiety in future global health crises.

Recommendations for immediate action were suggested under aspects of care to retain, reintroduce, and to remove. Maintaining access to essential reproductive and perinatal health services (37, 38), ensuring quality healthcare delivery (28, 31, 33), and giving perinatal mental health parity of esteem with physical health concerns (39), as well as providing specialist, tailored services for perinatal women (40–42), are recommended for retention as we recover and re-build services after the pandemic. Remote care should be retained (32, 33) but not at the expense of face-to-face consultation (24, 25, 35), nor should it be the dominant provision. Partners and family members, who women and birthing people want to be present, should be prioritised in healthcare settings (28, 31). Reinstating trust in the professional judgement of healthcare staff (32), ensuring adequate and timely communication amongst central NHS management, individual trusts, and healthcare professionals (31), and protecting staff from unnecessary redeployment (28, 31) are recommended for reinstation, whilst recognising the importance of social care being able to visit families rather than offering remote assessments and follow-up. Blanket policies made without considering demographic and accessibility variation should be ceased (30, 33, 56). Efforts should be made to investigate the long-term impacts of the COVID-19 pandemic on women, birthing people, and their families.

It is envisioned that this statement will provide a foundation for future research, policy implications, and service provision and care practices in perinatal mental health as we emerge from the pandemic, recover our healthcare systems and services, and build back a better provision for perinatal mental healthcare in the future. The services of the future must be resilient, adaptable, tensile, and plastic enough to weather future health system shocks when they inevitably arise—in order to provide the safest, most up-to-date, and best possible perinatal mental healthcare in the future.

## References

[1] Public Health England. COVID-19: epidemiology, virology, and clinical features. (2020). Available online at: https://www.gov.uk/government/publications/wuhan-novel-coronavirus-background-information/wuhan-novel-coronavirus-epidemiology-virology-and-clinical-features (accessed October 31, 2023).

[2] Elliott J Bodinier B Whitaker M Delpierre C Vermeulen R Tzoulaki I COVID-19 mortality in the UK biobank cohort: revisiting and evaluating risk factors. Eur J Epidemiol. (2021) 36(3):299–309. 10.1007/s10654-021-00722-y 33587202 PMC7882869

[3] WHO. WHO coronavirus (COVID-19) dashboard. (2021). Available online at: https://covid19.who.int/ (accessed August 24, 2021).

[4] Adhanom Ghebreyesus T. Addressing mental health needs: an integral part of COVID-19 response. World Psychiatry. (2020) 19(2):129–30. 10.1002/wps.20768 32394569 PMC7214944

[5] Jardine J Relph S Magee LA von Dadelszen P Morris E Ross-Davie M Maternity services in the UK during the coronavirus disease 2019 pandemic: a national survey of modifications to standard care. BJOG. (2021) 128(5):880–9. 10.1111/1471-0528.16547 32992408

[6] Hessami K Romanelli C Chiurazzi M Cozzolino M. COVID-19 pandemic and maternal mental health: a systematic review and meta-analysis. J Matern Fetal Neonatal Med. (2022) 35(20):4014–21. 10.1080/14767058.2020.1843155 33135523

[7] Racine N Eirich R Cooke J Zhu J Pador P Dunnewold N When the bough breaks: a systematic review and meta-analysis of mental health symptoms in mothers of young children during the COVID-19 pandemic. Infant Ment Health J. (2021) 43(1):36–54. 10.1002/imhj.21959 34962649 PMC9015533

[8] Benner AD Mistry RS. Child development during the COVID-19 pandemic through a life course theory lens. Child Dev Perspect. (2020) 14(4):236–43. 10.1111/cdep.12387 33230400 PMC7675461

[9] Liu S Fisher PA. Early experience unpredictability in child development as a model for understanding the impact of the COVID-19 pandemic: a translational neuroscience perspective. Dev Cogn Neurosci. (2022) 54:1–13. 10.1016/j.dcn.2022.101091 PMC8860470 35217299

[10] Thomas EY Anurudran A Robb K Burke TF. Spotlight on child abuse and neglect response in the time of COVID-19. Lancet Public Health. (2020) 5(7):e371. 10.1016/S2468-2667(20)30143-2 32619538 PMC7326432

[11] Qaderi K Khodavirdilou R Kalhor M Behbahani BM Keshavarz M Bashtian MH Abortion services during the COVID-19 pandemic: a systematic review. Reprod Health. (2023) 20(1):61. 10.1186/s12978-023-01582-3 37055839 PMC10098996

[12] Vousden N Ramakrishnan R Bunch K Morris E Simpson N Gale C Management and implications of severe COVID-19 in pregnancy in the UK: data from the UK obstetric surveillance system national cohort. Acta Obstet Gynecol Scand. (2022) 101(4):461–70. 10.1111/aogs.14329 35213734 PMC9111211

[13] Homer CE Leisher SH Aggarwal N Akuze J Babona D Blencowe H Counting stillbirths and COVID-19—there has never been a more urgent time. Lancet Glob Health. (2021) 9(1):10–1. 10.1016/S2214-109X(20)30456-3 PMC10011432 33212029

[14] Khalil A von Dadelszen P Draycott T Ugwumadu A O’Brien P Magee L. Change in the incidence of stillbirth and preterm delivery during the COVID-19 pandemic. JAMA. (2020) 324(7):705–6. 10.1001/jama.2020.12746 32648892 PMC7435343

[15] Sheen K Spiby H Slade P. Exposure to traumatic perinatal experiences and posttraumatic stress symptoms in midwives: prevalence and association with burnout. Int J Nurs Stud. (2015) 52(2):578–87. 10.1016/j.ijnurstu.2014.11.006 25561076

[16] Sheen K Goodfellow L Balling K Rymer J Weeks A Spiby H Which events are experienced as traumatic by obstetricians and gynaecologists, and why? A qualitative analysis from a cross-sectional survey and in-depth interviews. BMJ Open. (2022) 12(11):e061505. 10.1136/bmjopen-2022-061505 36410837 PMC9680185

[17] Slade P Balling K Sheen K Rymer J Goodfellow L Spiby H Work-related post-traumatic stress symptoms in obstetricians and gynaecologists: findings from INDIGO a mixed methods study with a cross–sectional survey and in-depth interviews. BJOG. (2020) 127(5):600–8. 10.1111/1471-0528.16076 31986555

[18] Manera K Hanson CS Gutman T Tong A. Consensus methods: nominal group technique. In: Liamputtong P, editor. Handbook of Research Methods in Health Social Sciences. Singapore: Springer (2019). p. 737–50. 10.1007/978-981-10-5251-4_100

[19] Fallon V Davies SM Silverio SA Jackson L De Pascalis L Harrold JA. Psychosocial experiences of postnatal women during the COVID-19 pandemic. A UK-wide study of prevalence rates and risk factors for clinically relevant depression and anxiety. J Psychiatr Res. (2021) 136:157–66. 10.1016/j.jpsychires.2021.01.048 33596462 PMC8635302

[20] UK Government. Prime Minister’s Statement on Coronavirus (COVID-19): 23 March 2020—GOV.UK. (2020). Available online at: www.gov.uk (accessed November 2, 2023).

[21] UK Government. Prime Minister’s Statement on Coronavirus (COVID-19): 11 May 2020—GOV.UK. (2020). Available online at: www.gov.uk (accessed November 2, 2023).

[22] UK Government. Prime Minister Confirms Move to Step 4—GOV.UK. (2021). Available online at: www.gov.uk (accessed November 2, 2023).

[23] Silverio SA Davies S Christiansen P Aparicio-García ME Bramante A Chen P A validation of the postpartum specific anxiety scale 12 item research short-form for use during global crises with five translations. BMC Pregnancy Childbirth. (2021) 21(1):1–12. 10.1186/s12884-021-03597-9 33557764 PMC7868877

[24] Jackson L De Pascalis L Harrold JA Fallon V Silverio SA. Postpartum women’s psychological experiences during the COVID-19 pandemic: a modified recurrent cross-sectional thematic analysis. BMC Pregnancy Childbirth. (2021) 21(625):1–16. 10.1186/s12884-021-04071-2 34530772 PMC8445650

[25] Jackson L Davies S Podkujko A Gaspar M De Pascalis L Harrold JA The antenatal psychological experiences of women during two phases of the COVID-19 pandemic: a recurrent, cross-sectional, thematic analysis. PLoS One. (2023) 18(6):1–24. 10.1371/journal.pone.0285270 PMC10249846 37289809

[26] Mateus V Cruz S Costa R Mesquita A Christoforou A Wilson CA Rates of depressive and anxiety symptoms in the perinatal period during the COVID-19 pandemic: comparisons between countries and with pre-pandemic data. J Affect Disord. (2022) 316:245–53. 10.1016/j.jad.2022.08.017 35964769 PMC9365708

[27] Mashayekh-Amiri S Asghari Jafarabadi M Montazeri M Fallon V Silverio SA Mirghafourvand M. Validation of the Iranian version of the postpartum specific anxiety scale 12-item research short-form for use during global crises (PSAS-IR-RSF-C). BMC Psychiatry. (2023) 23(511):1–9. 10.1186/s12888-023-04998-0 37452292 PMC10347867

[28] Montgomery E De Backer K Easter A Magee LA Sandall J Silverio SA. Navigating uncertainty alone: a grounded theory analysis of women’s psychosocial experiences of pregnancy and childbirth during the COVID-19 pandemic in London. Women Birth. (2023) 36(1):106–17. 10.1016/j.wombi.2022.05.002 PMC9110569 35610170

[29] Rimmer MP Al Wattar BH, UKARCOG Members. Provision of obstetrics and gynaecology services during the COVID-19 pandemic: a survey of junior doctors in the UK national health service. BJOG. (2020) 127(9) 1123–8. 10.1111/1471-0528.16313 32460422 PMC7283977

[30] Silverio SA Easter A Storey C Jurković D Sandall J, on behalf of the PUDDLES Global Collaboration. Preliminary findings on the experiences of care for parents who suffered perinatal bereavement during the COVID-19 pandemic. BMC Pregnancy Childbirth. (2021) 21(1) 840–53. 10.1186/s12884-021-04292-5 34937548 PMC8693591

[31] Bridle L Walton L Van Der Vord T Adebayo O Hall S Finlayson E Supporting perinatal mental health and wellbeing during COVID-19. Int J Environ Res Public Health. (2022) 19(3):1–12. 10.3390/ijerph19031777 PMC8835495 35162798

[32] Wilson CA Dalton-Locke C Johnson S Simpson A Oram S Howard LM. Challenges and opportunities of the COVID-19 pandemic for perinatal mental healthcare: a mixed-methods study of mental healthcare staff. Arch Women’s Ment Health. (2021) 24:749–57. 10.1007/s00737-021-01108-5 33830374 PMC8027292

[33] Pilav S Easter A Silverio SA De Backer K Sundaresh S Roberts S Experiences of perinatal mental healthcare among minority ethnic women during the COVID-19 pandemic in London: a qualitative study. Int J Environ Res Public Health. (2022) 19(4):1–15. 10.3390/ijerph19041975 PMC8872278 35206163

[34] Carson LE Azmi B Jewell A Taylor CL Flynn A Gill C Cohort profile: the eLIXIR partnership—a maternity–child data linkage for life course research in south London, UK. BMJ Open. (2020) 10(10):1–9. 10.1136/bmjopen-2020-039583 PMC7539583 33028561

[35] Hildersley R Easter A Bakolis I Carson L Howard LM. Changes in the identification and management of mental health and domestic abuse among pregnant women during the COVID-19 lockdown: regression discontinuity study. BJPsych Open. (2022) 8(4):1–12. 10.1192/bjo.2022.66 35657694 PMC9171064

[36] Loughnan SA Gautam R Silverio SA Boyle FM Cassidy J Ellwood D Multicountry study protocol of COCOON: continuing care in COVID-19 outbreak global survey of new, expectant, and bereaved parent experiences. BMJ Open. (2022) 12:1–12. 10.1136/bmjopen-2022-061550 PMC9445233 36691138

[37] Baxter AJ Geary RS Dema E Pérez RB Riddell J Willis M Contraceptive use and pregnancy planning in Britain during the first year of the COVID-19 pandemic: findings from a large, quasi-representative survey (Natsal-COVID). BMJ Sex Reprod Health. (2023) 49(4):1–14. 10.1136/bmjsrh-2022-201763 PMC10579517 36958823

[38] Romanis EC Parsons JA. Legal and policy responses to the delivery of abortion care during COVID-19. Int J Gynaecol Obstetr. (2020) 151:479–86. 10.1002/ijgo.13377 PMC9087790 32931598

[39] House of Commons. Mental health policy and services in England. (2023). Available online at: https://researchbriefings.files.parliament.uk/documents/CBP-7547/CBP-7547.pdf (accessed November 1, 2023).

[40] Alderdice F. What’s so special about perinatal mental health? J Reprod Infant Psychol. (2020) 38(2):111–2. 10.1080/02646838.2020.1734167 32183566

[41] Howard LM Khalifeh H. Perinatal mental health: a review of progress and challenges. World Psychiatry. (2020) 19(3):313–27. 10.1002/wps.20769 32931106 PMC7491613

[42] Department of Health and Social Care. Women’s health strategy for England. (2022). Available online at: https://www.gov.uk/government/publications/womens-health-strategy-for-england/womens-health-strategy-for-england (accessed November 1, 2023).

[43] Silverio SA De Backer K Brown JM Easter A Khazaezadeh N Rajasingam D Reflective, pragmatic, and reactive decision-making by maternity service providers during the SARS-CoV-2 pandemic health system shock: a qualitative, grounded theory analysis. BMC Pregnancy Childbirth. (2023) 23:1–15. 10.1186/s12884-023-05641-2 37210485 PMC10199292

[44] Landoni M Silverio SA Ionio C Giordano F. Managing children’s fears during the COVID-19 pandemic: strategies adopted by Italian caregivers. Int J Environ Res Public Health. (2022) 19(18):1–12. 10.3390/ijerph191811699 PMC9517545 36141968

[45] Hogg S Mayes G. Casting long shadows: The ongoing impact of the COVID-19 pandemic on babies, their families and the services that support them. London: The Institute of Health Visiting (2022).

[46] Fernandez Turienzo C Newburn M Agyepong A Buabeng R Dignam A Abe C Addressing inequities in maternal health among women living in communities of social disadvantage and ethnic diversity. BMC Public Health. (2021) 21(176):1–5. 10.1186/s12889-021-10182-4 33478445 PMC7817762

[47] Khan Z Vowles Z Fernandez Turienzo C Barry Z Brigante L Downe S Targeted health and social care interventions for women and infants who are disproportionately impacted by health inequalities in high-income countries: a systematic review. Int J Equity Health. (2023) 22(131):1–19. 10.1186/s12939-023-01948-w 37434187 PMC10334506

[48] Silverio SA Varman N Barry Z Khazaezadeh N Rajasingam D Magee LA Inside the “imperfect mosaic”: minority ethnic women’s qualitative experiences of race and ethnicity during pregnancy, childbirth, and maternity care in the United Kingdom. BMC Public Health. (2023) 23(2555):1–11. 10.1186/s12889-023-17505-7 38129856 PMC10734065

[49] Pilav S De Backer K Easter A Silverio SA Sundaresh S Roberts S A qualitative study of minority ethnic women’s experiences of access to and engagement with perinatal mental health care. BMC Pregnancy Childbirth. (2022) 22(421):1–13. 10.1186/s12884-022-04698-9 35585579 PMC9116695

[50] George-Carey R Memtsa M Kent-Nye FE Magee LA Oza M Burgess K Women’s experiences of early pregnancy loss services during the pandemic: a qualitative investigation. Women Birth. (2024). 10.1016/j.wombi.2023.12.004. [Epub ahead of print]. 38184398

[51] Worrall S Silverio SA Fallon V. The relationship between prematurity and maternal mental health in the first postpartum year. J Neonatal Nurs. (2023) 29(3):511–8. 10.1016/j.jnn.2022.10.002

[52] deMontigny F Verdon C Pierce T René C Landry I Corno G Vivre un décès périnatal en contexte de pandémie [Experiencing a perinatal death in the context of a pandemic]. Études Mort. (2023) 159(1):123–46. 10.3917/eslm.159.0123

[53] Silverio SA Memtsa M Barrett G Goodhart V Stephenson J Jurković D Emotional experiences of women who access early pregnancy assessment units: a qualitative investigation. J Psychosom Obstet Gynaecol. (2022) 43(4):574–84. 10.1080/0167482x.2022.2119958 36094423

[54] Silverio SA De Backer K Dasgupta T Torres O Easter A Khazaezadeh N On race and ethnicity during a global pandemic: an “imperfect mosaic” of maternal and child health services in ethnically-diverse south London, United Kingdom. eClinicalMedicine. (2022) 48(101433):1–10. 10.1016/j.eclinm.2022.101433 PMC9249549 35783482

[55] Balachandren N Barrett G Stephenson JM Yasmin E Mavrelos D Davies M Impact of the SARS-CoV-2 pandemic on access to contraception and pregnancy intentions: a national prospective cohort study of the UK population. BMJ Sex Reprod Health. (2022) 48(1):60–5. 10.1136/bmjsrh-2021-201164 34675063 PMC8550871

[56] Greenfield M. Lack of policy consideration for breastfeeding co-mothers in maternity services. Br J Midwifery. (2022) 30(9):526–30. 10.12968/bjom.2022.30.9.526

